# A Multifunctional and Highly Adaptable Reporter System for CRISPR/Cas Editing

**DOI:** 10.3390/ijms24098271

**Published:** 2023-05-05

**Authors:** Jochen M. Wettengel, Lea Hansen-Palmus, Sofiya Yusova, Lauren Rust, Sreya Biswas, Julien Carson, Junghyun Ryu, Benjamin N. Bimber, Jon D. Hennebold, Benjamin J. Burwitz

**Affiliations:** 1 Vaccine & Gene Therapy Institute, Oregon Health & Science University, Beaverton, OR 97006, USA; wettengel@tum.de (J.M.W.);; 2 Institute of Virology, Technical University of Munich/Helmholtz Zentrum München, 81675 München, Germany; 3 Division of Reproductive & Developmental, Oregon National Primate Research Center, Oregon Health & Science University, Beaverton, OR 97006, USA; ryuj@ohsu.edu (J.R.);; 4 Division of Genetics, Oregon National Primate Research Center, Oregon Health & Science University, Beaverton, OR 97006, USA; 5 Department of Obstetrics & Gynecology, Oregon Health & Science University, Portland, OR 97239, USA; 6 Division of Pathobiology & Immunology, Oregon National Primate Research Center, Oregon Health & Science University, Beaverton, OR 97006, USA

**Keywords:** CRISPR-Cas reporter system, Cas9 activity, saCas9, spCas9, asCas12a, HDR, NHEJ

## Abstract

CRISPR/Cas systems are some of the most promising tools for therapeutic genome editing. The use of these systems is contingent on the optimal designs of guides and homology-directed repair (HDR) templates. While this design can be achieved in silico, validation and further optimization are usually performed with the help of reporter systems. Here, we describe a novel reporter system, termed BETLE, that allows for the fast, sensitive, and cell-specific detection of genome editing and template-specific HDR by encoding multiple reporter proteins in different open-reading frames. Out-of-frame non-homologous end joining (NHEJ) leads to the expression of either secretable NanoLuc luciferase, enabling a highly sensitive and low-cost analysis of editing, or fluorescent mTagBFP2, allowing for the enumeration and tissue-specific localization of genome-edited cells. BETLE includes a site to validate CRISPR/Cas systems for a sequence-of-interest, making it broadly adaptable. We evaluated BETLE using a defective moxGFP with a 39-base-pair deletion and showed spCas9, saCas9, and asCas12a editing as well as sequence-specific HDR and the repair of moxGFP in cell lines with single and multiple reporter integrants. Taken together, these data show that BETLE allows for the rapid detection and optimization of CRISPR/Cas genome editing and HDR in vitro and represents a state-of the art tool for future applications in vivo.

## 1. Introduction

Genome editing systems using clustered regularly interspaced short palindromic repeats (CRISPR) have revolutionized genetic biology and become some of the most promising technologies in molecular medicine [1]. The technology comprises sequence-specific guide RNAs (gRNAs) that assemble with the CRISPR-associated (Cas) endonucleases. These ribonucleoproteins (RNP) induce breaks in close proximity to the complementary sequence on a target DNA (or RNA) [2].

Currently, the most studied Cas enzymes are Cas9 endonucleases, which were originally discovered in Streptococcus pyogenes (spCas9); however, orthologues with diverse characteristics have been found in other bacterial species such as Staphylococcus aureus (saCas9) [3,4]. In addition to Cas9 enzymes, Cas12a (formerly Cpf1) and CasΦ endonucleases in particular are now emerging as strong pre-clinical candidates due to their reported reduction in off-target effects, absence of transactivating CRISPR RNA, smaller size, and ability to process their own CRISPR RNAs (crRNAs) without the requirement of additional proteins [5,6].

In molecular medicine, CRISPR/Cas technology is currently being studied for the treatment of multiple chronic viral infections, cancers, and genetic disorders [7,8,9]. In these settings, the most prominent genetic modifications induced by CRISPR/Cas are: (1) double-stranded DNA breakage, followed by stochastic repair through non-homologous end joining (NHEJ), leading to frameshift mutations and/or gene disruption, and (2) gene repair or reconstruction via homology-directed repair (HDR) using a sequence-specific repair template [10]. Both modifications represent potential genetic medical treatments, but further research must be undertaken in order to optimize genome editing efficiency, specificity, and safety before clinical use. 

Indeed, current approaches using viral vector or liposomal particle transfer are favored for their efficient and tissue-specific delivery, but their validation is contingent upon suitable reporter systems in vitro and in vivo. To date, several CRISPR/Cas reporter systems have been established to answer specific scientific questions. Most of these reporter systems focus on the optimization of CRISPR/Cas double-strand breaks followed by NHEJ, in which induced frame-shift mutations turn on a downstream single [11,12,13,14] or dual [15,16,17] out-of-frame fluorescent protein. For the analysis of HDR, reporters have been designed that incorporate a defective fluorescent protein [14,17], a split fluorescent protein [18], or antibiotic resistance genes [19,20]. Although the readout of these systems via fluorescence microscopy, flow cytometry, or antibiotic selection allows for the counting and enrichment of edited cells, high-throughput assays rely on fast and sensitive read-outs such as luciferase [21,22]. However, emerging CRISPR/Cas technologies and future in vivo reporter animal models are dependent on the analysis of multiple aspects of CRISPR/Cas editing in a single combinatory reporter system.

Here, we summarize an all-in-one reporter system named BETLE (bivalent enhanced traffic light editing) that can be used to optimize and analyze CRISPR/Cas editing, including both NHEJ and HDR efficiencies. With this system, cell- and tissue-specific CRISPR/Cas-mediated gene disruption can be easily analyzed via the editing of a constitutively expressed fluorescent mCherry. Furthermore, mCherry is followed by a P2A site and a sequence-of-interest (SOI) which can be easily adapted to represent any genomic target, provided it can be codon-optimized to remove out-of-frame stop codons. Most importantly, the frameshift mutations caused by genome editing lead to fluorescent or secreted luminescent readouts, allowing for cell- and tissue-specific fluorescence microscopy and flow cytometry while also providing a global editing analysis by a secretable luciferase in supernatant in vitro or in the bodily fluids of a future in vivo reporter animal models [23].

## 2. Results

### 2.1. Design of the Bivalent Enhanced Traffic Light Editing Reporter

**Figure 1:**
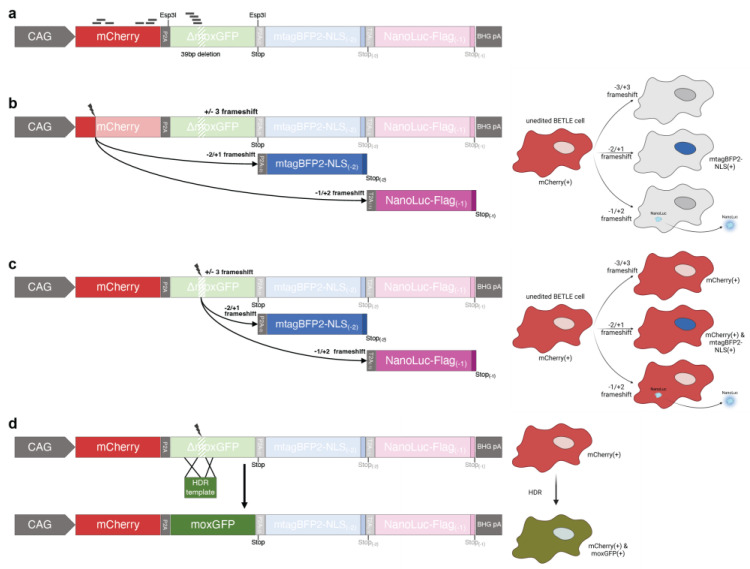
Schematic showing the BETLE reporter system. (**a**) The unedited BETLE reporter consists of an mCherry P2A-linked to the SOI (here ΔmoxGFP with a 39 bp deletion), which can be exchanged through individual Esp3I cleavage sites, followed by an (−2 bp) out-of-frame P2A-linked mTagBFP2 and a (−1 bp) out-of-frame T2A-linked NanoLuc luciferase cassette for editing outcome prediction using fluorescence and luminescence readouts. Location of spCas9 guide sequences used in this report are indicated with lines. Further, different fluorescence and luminescence outcomes are indicated via a sequence and cell schematics upon CRISPR-mediated frameshift in the (**b**) mCherry region, (**c**) ΔmoxGFP region, and (**d**) ΔmoxGFP region, followed by a moxGFP HDR.

Based on previous reporter systems, we designed a new Bivalent Enhanced Traffic Light Editing (BETLE) reporter with two different readouts: fluorescence and luminescence. We improved upon the original reporters by incorporating a combination of possible readouts to specifically measure (1) sequence-specific CRISPR/Cas editing, including gene disruption, frameshift mutations, and HDR; (2) the spatial detection of CRISPR/Cas edited cells; and (3) overall levels of NHEJ using a non-invasive analysis of secreted luciferase. We achieved this by linking mCherry with an exchangeable SOI, separated by a P2A site and followed by fluorescent and luminescent cassettes also separated by individual 2A cleavage sites in distinct open-reading frames (Figure 1a). We engineered two Esp3I sites with different overhangs in the flanking P2A sites to allow for easy and rapid switching of the SOI for editing. To demonstrate functionality, we chose a codon-optimized ΔmoxGFP with a 39-base-pair (bp) deletion in the active site, allowing for assessment of HDR efficiency [24]. 

In its native state, this configuration of the BETLE reporter constitutively transcribes and translates mCherry-P2A-ΔmoxGFP under the CAG promoter (Figure 1a). The mCherry-P2A-ΔmoxGFP ORF is followed by both an N−2 ORF containing a P2A-mTagBFP2 cassette and an N−1 ORF containing a T2A-NanoLuc cassette [25,26]. Thus, the out-of-frame CRISPR/Cas editing of either mCherry or the SOI leads to readouts that are intracellular (mTagBFP2 with a nuclear localization signal (NLS)) and extracellular (secreted, FLAG-tagged NanoLuc), providing the user with multiple options for measuring editing efficiency and specificity (Figure 1b,c). In addition, the efficiency of successful HDR can be assessed by detecting both mCherry and moxGFP (Figure 1d).

We cloned our reporter sequence into a piggyBac transposon plasmid and transfected HEK293A cells with this plasmid in addition to the piggyBac transposase for stable genome integration [27]. We then expanded and selected these cells with Geneticin (G418) for two weeks prior to the flow cytometric sorting of the mCherry(+) cells. These sorted cells were then expanded and are referred to hereafter as BETLE-Pop (population).

We then selected mCherry-specific spCas9 gRNAs predicted for high-efficiency editing (Figure 1a and Table S1, reporter sites A–C) and multiple overlapping spCas9, saCas9, and Cas12a gRNAs targeting ΔmoxGFP at the deletion site to confirm that editing leads to the anticipated changes in reporter function (Table S1, reporter site D). Two ΔmoxGFP spCas9 gRNAs containing nucleotide mismatches were used as controls (Table S1, reporter site D, mismatches marked in red lower-case).

### 2.2. Identification of Highly Efficient gRNAs for CRISPR/Cas Editing Using BETLE Reporter

**Figure 2:**
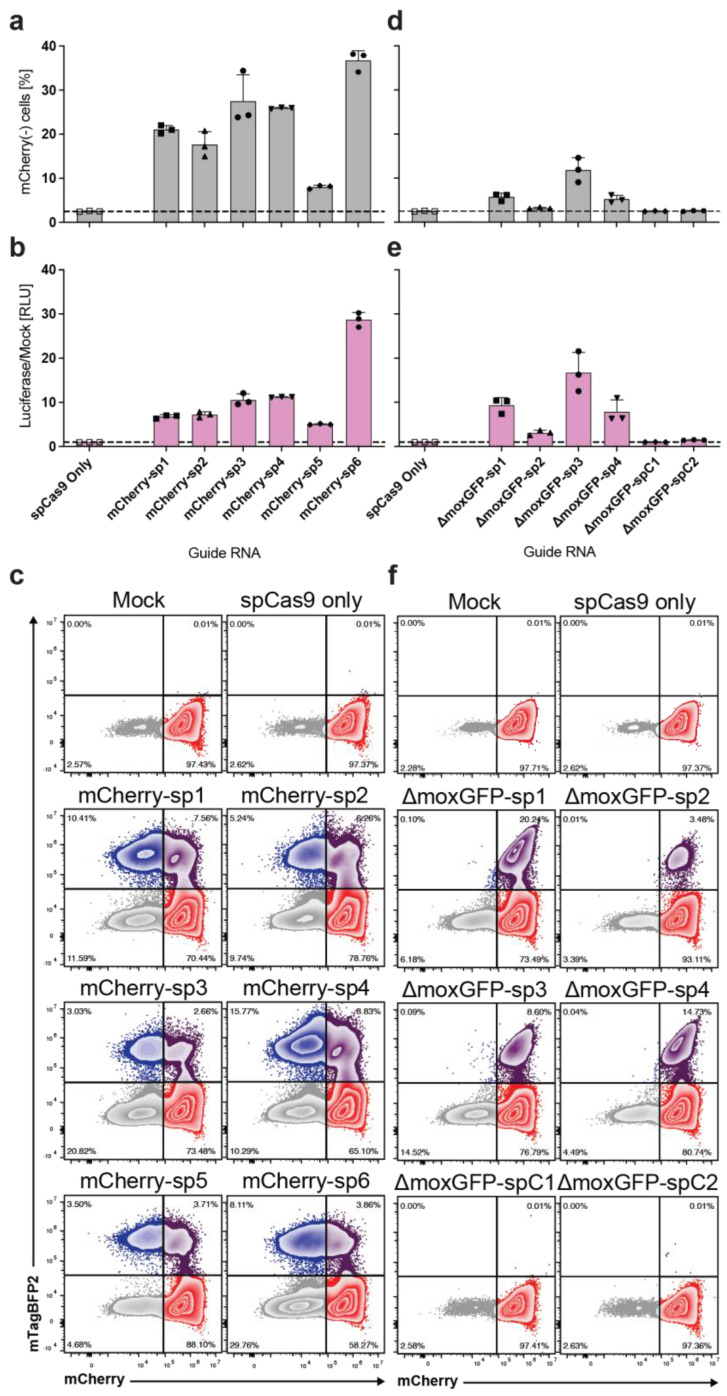
Identification of highly efficient gRNAs for SpCas9-mediated CRISPR editing. HEK293A cells stably expressing the BETLE reporter (BETLE-Pop) were transfected with plasmids expressing SpCas9 as well as gRNAs. Transfection with plasmid containing only SpCas9 and without plasmid (Mock) served as controls. (**a**) Frequency of mCherry(−) cells was determined via FACS analysis. (**b**) N−1 editing of mCherry was detected by quantifying NanoLuc luciferase activity in the supernatant of transfected cells. Dotted lines indicate level of Mock-treated cells. (**c**) N−2 editing of mCherry was analyzed via FACS analysis. (**d**–**f**) The same as (**a**–**c**) but for gRNAs targeting ΔmoxGFP.

In order to demonstrate that BETLE can identify gRNAs with the highest efficiency, we tested the six mCherry-specific spCas9 gRNAs (mCherry-sp1–sp6) for mCherry gene disruption (Table S1, reporter sites A–C). We transfected BETLΕ-Pop with DNA plasmids expressing both spCas9 and gRNAs and analyzed the frequency of mCherry(−) cells (Figure 2a). We found that the gRNA mCherry-sp6 was the most efficient, with >30% mCherry(−) cells. While this readout is not suited for high-throughput screenings, we assessed N−1 editing by measuring the secreted NanoLuc luciferase from transfected cells and found a strong luminescence signal in the supernatant of all cells targeted by gRNAs but not in the supernatant of cells treated with spCas9 alone (no gRNA). Here, we again confirmed that gRNA mCherry-sp6 obtained the highest editing efficiency (N−1), demonstrating the use of BETLE in high-throughput (N−1 editing) gRNA screenings (Figure 2b). In order to identify cells with N−2 editing, we analyzed the cells on a flow cytometer and found between 5.69% and 24.6% mTagBFP2(+) cells (Figure 2c). Notably, in this experiment, gRNA mCherry-sp4 had the highest frequency of mTagBFP2(+) cells, indicating gRNA-specific preferences for N−1 or N−2 editing. Since the spatial data of the N−1 NanoLuc ORF editing may also be of interest, we showed that NanoLuc-expressing cells can be stained using a fluorescent-labeled anti-FLAG-antibody (Figure S1). 

To identify highly efficient gRNAs for ΔmoxGFP, we repeated the previous experiments with the four ΔmoxGFP-specific spCas9 gRNAs (gRNA-ΔmoxGFP-sp1–sp4) and included the two control spCas9 gRNAs (gRNA-ΔmoxGFP-spC1–spC2) (Figure 1c, Table S1, reporter site D). We identified gRNA ΔmoxGFP-sp3 as the best N−1 editor with the highest NanoLuc secretion, and gRNA ΔmoxGFP-sp1 as the best N−2 editor with the highest number of mTagBFP2(+) cells, again indicating the differential N−1 and N−2 preferences of each gRNA (Figure 2d–f). 

In order to further analyze the edited cells, we performed fluorescence microscopy and showed mTagBFP2(+) nuclei, indicating that the localization of cell- and tissue-specific editing is possible (Figure S2). Moreover, we monitored NanoLuc expression kinetics longitudinally with a peak, sustained luminescence by day 6, showing stable editing (Figure S3). 

Finally, to control for transfection efficiencies, we included a puromycin selection marker on plasmids expressing Cas9 and sgRNA. A short 48 h selection with puromycin (10 μg/mL), beginning 24 h post transfection, revealed a highly purified population of edited cells. This selection was even able to detect small populations of edited cells following treatment with control sgRNA with mismatched sequences (Figure S4).

### 2.3. Analysis of CRISPR/Cas Editing in BETLE Reporter Cells with Single and Multiple Integrants

**Figure 3:**
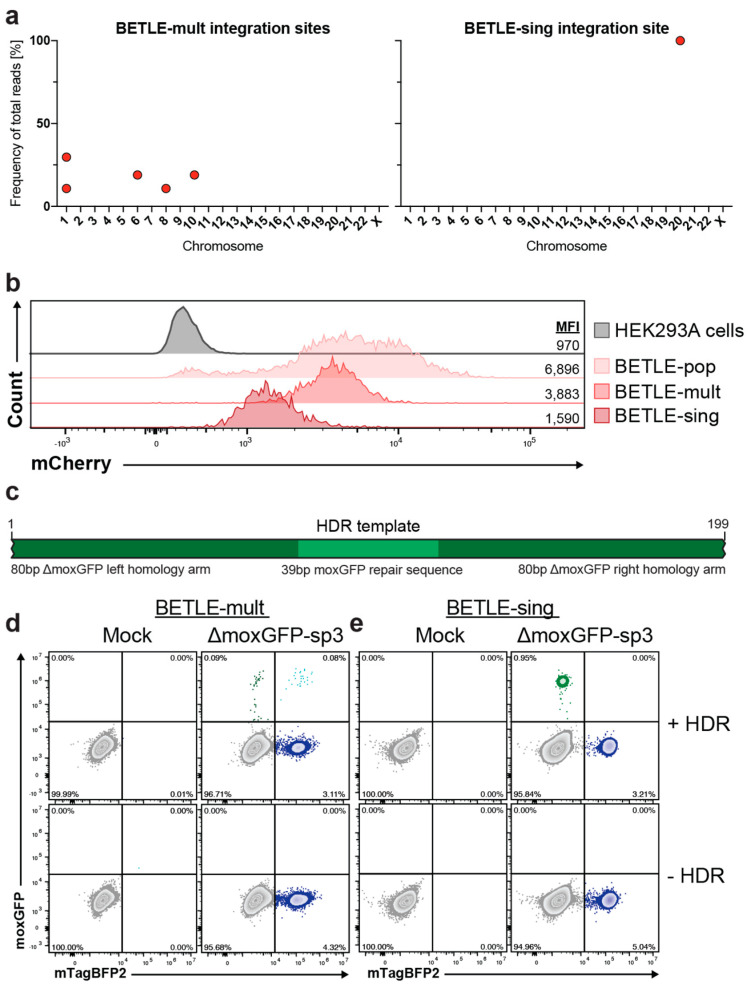
CRISPR/Cas editing in cells with single and multiple BETLE reporter integrants. (**a**) Single-cell clones BETLE-mult and BETLE-sing were isolated from BETLE-Pop and analyzed for their number of BETLE reporter integrants. (**b**) mCherry expression in different BETLE reporter cell lines via FACS analysis. (**c**) Design of a 199 bp HDR template encoding the missing 39 bp of ΔmoxGFP between two 80 bp homology arms. (**d**) BETLE-mult and (**e**) BETLE-sing cells were co-transfected with the most-efficient gRNA ΔmoxGFP-sp3 and the HDR template. ΔmoxGFP repair was analyzed via FACS analysis.

To further validate the consistency and robustness of the BETLE reporter, we derived clones from BETLE-Pop via single-cell dilution. We performed a previously published modified tagmentation PCR assay to define the number and location of all integrants in the clones and selected both a clone with multiple integrants (BETLE-mult) and a clone with a single integrant (BETLE-sing) (Figure 3a) [28]. This assay confirmed five integration sites in BETLE-mult across multiple chromosomes and a single integration site in BETLE-sing on chromosome 20 (Table S2). We analyzed the mCherry mean fluorescence intensities (MFI) of the clones and compared it to the BETLE-Pop and non-transfected HEK293A cells. As expected, we saw higher mCherry expression in the BETLE-mult compared to BETLE-sing, as well as a homogeneous mCherry expression profile across both BETLE clones (Figure 3b). 

According to previous studies [29], we next designed a 199 bp ssDNA HDR template with 80 bp homology arms on each side and the missing 39 bp of ΔmoxGFP (Figure 3c) to achieve a high HDR efficiency. We then co-transfected each BETLE clone individually with the most efficient gRNA from the previous experiment (gRNA ΔmoxGFP-sp3) (Figure 2d) in combination with the HDR template. Using flow cytometry, we quantified the frequency of the repaired moxGFP(+) cells (Figure S5). Notably, BETLE-mult exhibited moxGFP(+)/mTagBFP2(+) cells (Figure 3d), indicating a mixture of NHEJ and HDR amongst the integrants of some edited cells. In contrast, we did not find these moxGFP(+)/mTagBFP2(+) cells in BETLE-sing via either flow cytometry (Figure 3e) or fluorescence microscopy (Figure S6), confirming the single genome integrant of this clone, which undergoes either NHEJ or HDR. These results show that CRISPR/Cas editing and HDR do not necessarily occur in all cellular integrants, highlighting the strength and potential test options of this reporter system.

**Figure 4:**
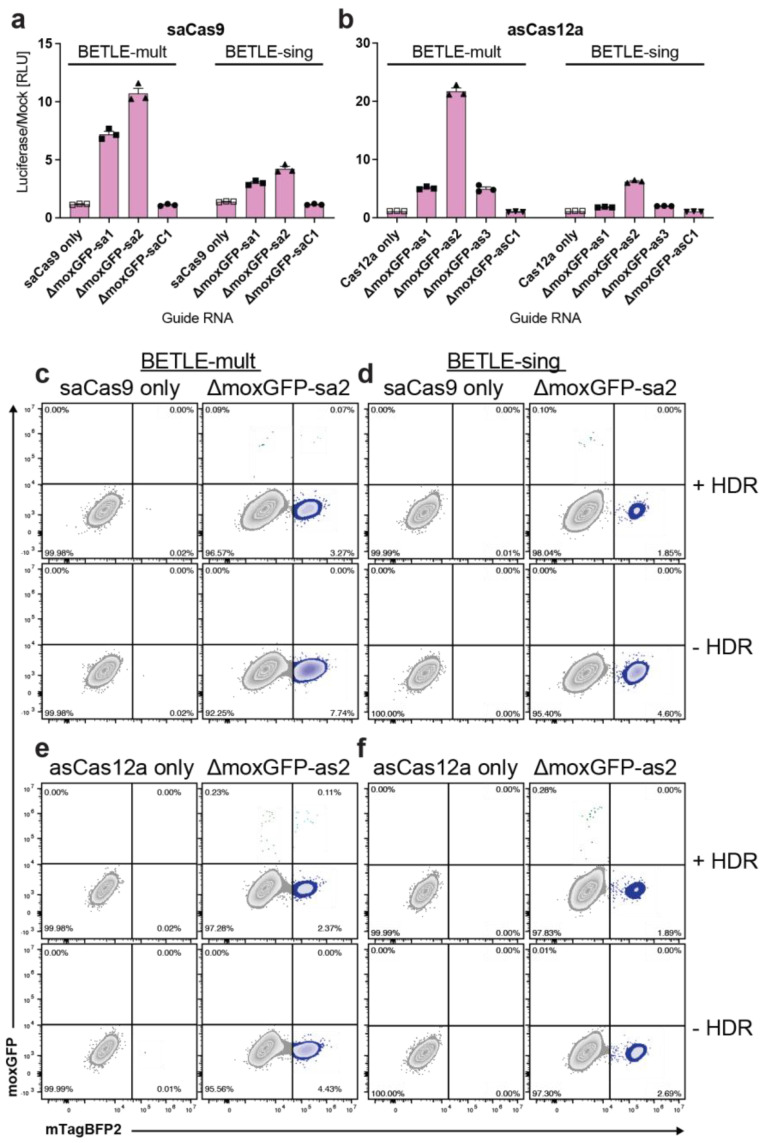
Screening of different Cas endonucleases and gRNAs for editing and HDR efficiency. BETLE-mult and BETLE-sing cells were transfected with plasmids expressing (**a**) saCas9 or (**b**) asCas12a and ΔmoxGFP gRNAs. Editing was measured by quantifying NanoLuc luciferase activity. To analyze HDR-mediated target repair with different Cas enzymes, BETLE-mult and BETLE-sing cells were co-transfected with SaCas9 gRNAs and HDR template (**c**,**d**) or asCas12a gRNAs and HDR template (**e**,**f**). Target repair was analyzed via FACS analysis.

Next, we screened different Cas enzymes and gRNAs for their genome-editing efficiency using NanoLuc (N−1 editing) as a readout. BETLΕ-mult and BETLE-sing were transfected with DNA plasmids expressing either saCas9 or *Acidaminococcus* sp. Cas12a (asCas12a) in addition to ΔmoxGFP-specific gRNAs (ΔmoxGFP-sa1–sa2 or ΔmoxGFP-as1–as2). Again, gRNAs with mismatches were used as negative controls (ΔmoxGFP-saC1 and ΔmoxGFP-asC1). As expected, we could identify strong differences in luciferase expression based on the combination of gRNAs and Cas endonucleases (Figure 4a,b). Similar to the previous experiment, HDR efficiency was tested using gRNAs that yielded the highest luminescence in BETLE-mult and BETLE-sing. Successful moxGFP(+) HDR was observed in both cell lines (Figure S7), but BETLE-mult again exhibited both moxGFP(+)/mTagBFP2(+) and moxGFP(+)/mTagBFP2(−) cells (Figure 4c,e), while BETLE-sing showed only moxGFP(+)/mTagBFP2(−) cells via flow cytometry (Figure 4d,f) and fluorescence microscopy (Figure S8), supporting the previous data.

### 2.4. Sequence Confirmation of BETLE Reporter Editing

**Figure 5:**
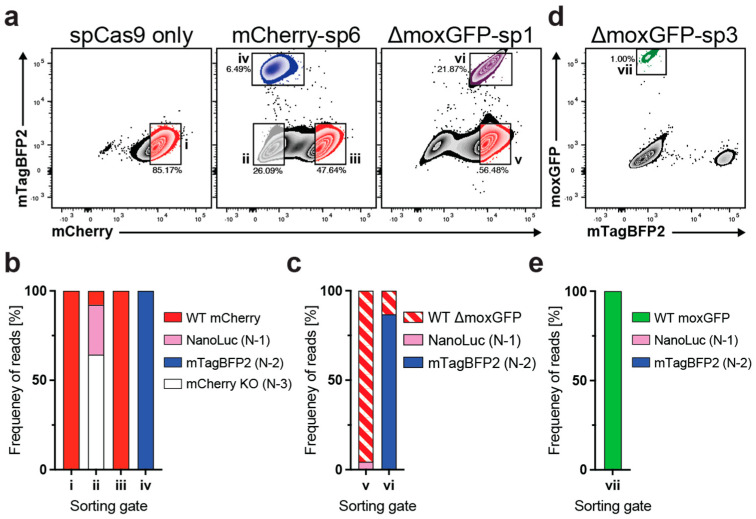
Sequence confirmation of BETLE reporter editing. (**a**) BETLE-sing was transfected with spCas9 only, mCherry-sp6, or ΔmoxGFP-sp1, and cells were sorted into the indicated populations i–vi. (**b**,**c**) Genomic DNA was extracted from sorted populations i–vi, and the gRNA target sites were sequenced. (**d**) BETLE-sing was transfected with ΔmoxGFP-sp3 and the HDR template and cells were sorted into the indicated population vii. (**e**) Genomic DNA was extracted from sorted population vii, and the gRNA target sites were sequenced.

Having shown the successful expression of reporter genes in the N−1 and N−2 ORFs, we next analyzed the targeted DNA sequences in edited cells. Given the challenges of sequencing edited cells with multiple integrants, we focused on BETLE-sing cells. We transfected BETLΕ-sing cells with DNA plasmids expressing spCas9 only or both spCas9 and gRNAs targeting mCherry or ΔmoxGFP and sorted the edited cells based on their fluorescent profile. While cells transfected with spCas9 only had a single population (i), the cells transfected with gRNA mCherry-sp6 showed three (ii, iii, and iv) distinct populations, and the cells transfected with ΔmoxGFP-sp3 showed two (v and vi) distinct populations (Figure 5a). We then extracted genomic DNA, PCR-amplified the target sites, and sequenced the PCR products. 

All cell phenotypes correlated with the predicted DNA sequence within the cells. Unedited BETLE-sing cells transfected with spCas9 only (i) exhibited a complete conservation of the target sequence in 100% of the reads, with no deletions or insertions (Figure 5b). However, while unedited BETLE-sing cells treated with gRNA mCherry-sp6 were mCherry(+) and showed conservation of the target sequence in 100% of the reads (iii), edited BETLE-sing cells were mCherry(−) and stratified, as expected, into populations that were either mTagBFP2(+) (iv) or mTagBFP2(−) (ii) (Figure 5b). The mTagBFP2(+) population (iv) exhibited 100% N−2 insertions or deletions, while population (ii) showed both N−1 out-of-frame (27.9%) and also N−3 in-frame (64.4%) deletions and insertions.

The BETLE-sing cells treated with gRNA moxGFP-sp1 exhibited two main populations expressing mCherry as expected, mTagBFP2(−) (v) or mTagBFP2(+) (vi) (Figure 5a). Population (v) contained 4.5% N−1 insertions or deletions. However, since population (v) consisted of both non-edited and edited cells, the majority of sequences (95.5%) showed no insertions or deletions (Figure 5c). As expected, population (vi) contained 86.9% N−2 insertions or deletions, leading to mTagBFP2 expression. Finally, we sequenced DNA from moxGFP(+) BETLE-sing cells (vii) treated with gRNA moxGFP-sp3 and the ssDNA HDR template (Figure 5d), finding that 100% of the sequences were repaired moxGFP (Figure 5e). Taken together, these data confirmed that the BETLE reporter can identify DNA editing, without the need for sequencing.

## 3. Discussion and Conclusions

In order to translate genome editing into the clinic, the efficiency of editors and delivery vectors must be determined using suitable reporter systems. While most current genome editing reporters measure distinct outcomes such as gRNA efficiency, NHEJ, or HDR individually, none of them combine all these readouts as in the BETLE reporter. Indeed, the BETLE reporter can be used to analyze gene disruption, out-of-frame mutations, and HDR in a single system. We accomplished this by expressing mCherry P2A-linked to an SOI followed by out-of-frame encoded reporter proteins. Notably, the SOI is broadly adaptable but must be codon-optimized to remove stop-codons (TAA, TAG, and TGA) in all frames due to the subsequent out-of-frame encoded reporter genes. Through the flanking Esp3I restriction sites, the SOI is easily exchangeable such that the BETLE reporter can also be used for high-throughput screening. Indeed, instead of screening a complete optimized gene sequence, reporters with short gRNA-specific sequences could be cloned and pre-screened for editing efficiency. 

For our BETLE reporter, we chose mCherry and mTagBFP2 as fluorescence reporters since these proteins, in addition to our SOI ΔmoxGFP, exhibit robust fluorescence, broad stability, and share neither high emission overlap nor sequence homology, minimizing non-specific editing and recombination. We also attached a nuclear localization signal to mTagBFP2 to concentrate fluorescence in the nucleus and improved the fluorescence signal-to-noise ratio. Moreover, we selected NanoLuc as our luciferase reporter protein due to its small size, high luminescence efficiency, and ability to be secreted. By linking a FLAG-tag to the C-terminus of NanoLuc, antibody staining can be used to detect and distinguish between N−1 and N−2 frame-shifts on a single-cell level. Since our data clearly showed that gRNAs exhibit different preferences for out-of-frame deletions and insertions, this phenomenon can be further and more easily analyzed using this BETLE reporter or a slightly adapted version. Indeed, potentially advantageous adaptations to the BETLE reporter include the exchange of the mTagBFP2 by a tagged second luciferase gene or the replacement of NanoLuc by another fluorescence gene to obtain a complete luminescence or fluorescence reporter, respectively. 

Finally, our design of the BETLE reporter was specifically focused on the generation of transgenic animal models for the treatment of genetic disorders, cancer, and infectious diseases, potentially requiring the targeting of single, dual, or multiple sites. Since our BETLE reporter was used successfully in single and multiple integrated cell lines to show different editing outcomes, animal models with single, dual, or multiple integrations of the BETLE reporter into the germline may represent a powerful tool for clinical translation. Overall, this novel BETLE reporter system allows for the rapid detection and optimization of CRISPR/Cas genome editing and HDR in vitro and represents a state of the art tool for future in vivo analysis in transgenic animals.

## 4. Materials and Methods

### 4.1. BETLE Reporter Design and Synthesis

The BETLE reporter cassette was synthesized (GeneArt, Regensburg, Germany) and cloned into a piggyBac transposon-encoding plasmid. This plasmid, termed pB_CAG_BETLE_Neo, was then transfected with a plasmid-encoding piggyBac transposase into HEK293A cells using Lipofectamine 3000 (ThermoFisher Scientific, Waltham, MA, USA) according to the manufacturer’s instructions. For the generation of a cell population with integrated BETLE reporter expression (BETLE-pop), HEK293A cells were seeded and transfected on a 24-well plate, followed by an expansion on a 6-well plate for 3 days and antibiotic selection for another 14 days with Geneticin (G418) (ThermoFisher Scientific, Waltham, MA, USA). The complete sequence of the BETLE reporter is available (GenBank accession #OK480061).

### 4.2. Cloning of BETLE Reporter Cell Lines

Clonal cell lines with multiple (BETLE-mult) or single (BETLE-sing) BETLE integrants were established by performing a single-cell dilution of BETLE-pop cell line in a 96-well plate.

### 4.3. Editing of BETLE Reporter Cell Lines

BETLE reporter cells were edited following transfection with CRISPR/Cas encoding plasmids LentiCRISPRv2 (Addgene #98290), pX601 (Addgene #107055), or pY30 (Addgene #84745) with specific gRNAs. gRNAs were designed using http://crispor.tefor.net/ (accessed on 18 September 2021). The cells were seeded on a 24-well plate and transfected with the plasmids, using Lipofectamine 3000 according to manufacturer’s instructions. For HDR, the respective plasmid was mixed with the 199 bp ssDNA HDR template (synthesized by IDT as an Ultramer) in a DNA mass ratio of 1:1, using Lipofectamine 3000 according to manufacturer’s instructions.

### 4.4. NanoLuc-Luciferase Measurement

Luciferase activity was determined by transferring 10 µL of supernatant into a white 96-well plate and measuring luminescence on a Tecan Infinite 200 plate reader after adding 100 µL of PBS-T (0.1% Tween 20) containing a 1:1000 dilution of 1 mM Coelenterazine N (dissolved in acidified methanol) stock solution.

### 4.5. Fluorescence Microscopy

Fluorescence microscopy was performed on live cells using a Leica DMi8 fluorescence microscope with filter cubes DAPI, FITC, and TXR.

### 4.6. Identification of BETLE Integration Sites

Transgene integration sites were mapped using enhanced-sensitivity tagmentation-assisted PCR (esTag-PCR) [28]. The initial tagmentation step was performed with 100 ng of gDNA in a tagmentation reaction that included: 25 µL TD buffer, 2.5 µL Ilumina TDE1 enzyme, 100 ng DNA, and H2O to a final volume of 50 µL. After preheating the thermocycler to 58 °C, the reaction mixture was incubated for 5 min at 58 °C, followed by a 10 °C hold. The fragmented DNA was purified using Ampure XP beads with a 0.8 bead to sample ratio and eluted into 30 µL H_2_O. Following tagmentation, the first exponential PCR was then performed using 25 µL KAPA HiFi HotStart ReadyMix (Kapa Biosystems KK2602), 1.25 µL of each primer at 10 µM (see “PCR1” Table S3), and 21.25 µL tagmentation product. The following PCR conditions were used: 95 °C for 3 min, followed by 30 cycles of: 98 °C for 30 s, 63 °C for 30 s, and 72 °C for 1 min, followed by 72 °C for 10 min and a 4 °C hold. A secondary nested PCR was then performed using 12.5 µL KAPA HiFi HotStart ReadyMix, 1.25 µL of index primer at 10 µM, 0.75 µL of each transgene-specific primer at 10 µM (see “PCR2” Table S3), 2 µL of the first-round PCR, and 7.5 µL H_2_O. PCR conditions for the nested PCR were 98 °C for 45 s, followed by 10 cycles of 98 °C for 20 s, 54 °C for 30 s, and 72 °C for 20 s, ending with 72 °C for 1 min and a 4 °C hold. The second PCR added a unique index to each sample. The PCR reaction was cleaned using Ampure XP beads at a 0.8 bead-to-sample ratio. Purified samples were quantified using a Qubit Fluorometer (Invitrogen, Waltham, MA, USA). The resulting sequence libraries were sequenced on an Illumina MiSeq instrument using single-end 150 bp reads. The resulting FASTQ data were quality trimmed using Trimmomatic [30]. Next, the reads were filtered to retain only reads or read pairs in which at least one read contained the terminal 15 mer nucleotide sequence from either terminal end of the transgene, allowing for an edit-distance of 2. This was performed using the tool PrintReadsContaining, which is part of the DISCVR-seq software package (https://github.com/bimberlab/discvrseq (accessed on 15 February 2022)). The passing reads were aligned to the reference genome GRCh38.p13 (release 98; assembly ID GCA_000001405.28) using BWA-mem [31,32]. The location and orientation of integration events was then mapped using the tool IntegrationSiteMapper, which is also part of the DISCVR-seq software package.

### 4.7. Flow Cytometry

BETLE reporter cells were stained with fixable live/dead amine reactive dye (ThermoFisher Scientific) with a recombinant anti-FLAG antibody (Miltenyi Biotec, Bergisch Gladbach, Germany) for 20 min. The cells were then washed twice in FACS buffer (PBS + 2% fetal bovine serum) and collected live on an LSR2 (Becton-Dickinson, Franklin Lakes, NJ, USA). For FLAG staining, the cells were fixed in a final concentration of 2% PFA for 20 min. The cells were then washed twice in permeabilization buffer (0.1% saponin in FACS buffer), stained with anti-FLAG-APC (Miltenyi Biotec) for 20 min, and again washed twice in a permeabilization buffer. The cells were then collected on the LSR2.

### 4.8. Illumina Sequencing of BETLE Reporter

Cell populations were sorted by fluorescence profile on an FACSAria (Becton-Dickinson). Genomic DNA was extracted from cells using the All-prep DNA/RNA extraction kit (Qiagen, Hilden, Germany). The pertinent regions of the genome were PCR-amplified using the forward primer 5′-TCCTGGGCAACGTGCTGGTTATTG-3′ and the reverse primer 5′-GGTCTTGGAGCCGTACAGAAAGGAG-3′. Gel-purified DNA was normalized to 5 ng/μL using water and processed through the Twist Bioscience Protocol for library preparation as per the manufacturer’s instructions. Libraries were sequenced on an Illumina MiSeq, using paired-end 250 bp reads. The raw FASTQ reads were quality trimmed using Trimmomatic.21 Paired reads were merged using FLASH [33]. Merged reads were aligned to the reference plasmid sequence using BWA-mem.22,23. We used the tool PrintReadBackedHaplotypes, which is a readily available part of the DISCVR-seq software package (https://github.com/bimberlab/discvrseq (accessed on 15 February 2022)), to identify and count all unique haplotypes over the sgRNA regions. The interval sequences analyzed (using GenBank accession #OK480061) were:

mCherry region A: 1910 bp–2009 bp
mCherry region C: 2368 bp–2467 bp
ΔmoxGFP region D: 2666 bp–2765 bp

## Data Availability

The BETLE reporter sequence is available through GenBank accession #OK480061 and Addgene plasmid #177864. The primary data and additional materials reported in this study are available from the corresponding author upon request.

## Supplementary Materials

The following supporting information can be downloaded at: https://www.mdpi.com/article/10.3390/ijms24098271/s1.
