# Supplementary material for: A Multifunctional and Highly Adaptable Reporter System for CRISPR/Cas Editing
Source: Int J Mol Sci. 2023 May 5;24(9):8271. doi: 10.3390/ijms24098271 (PMC10179647; doi:10.3390/ijms24098271)
Supplement: Supplementary file 1 [file ijms-24-08271-s001.zip › ijms-2354464-SI.pdf]

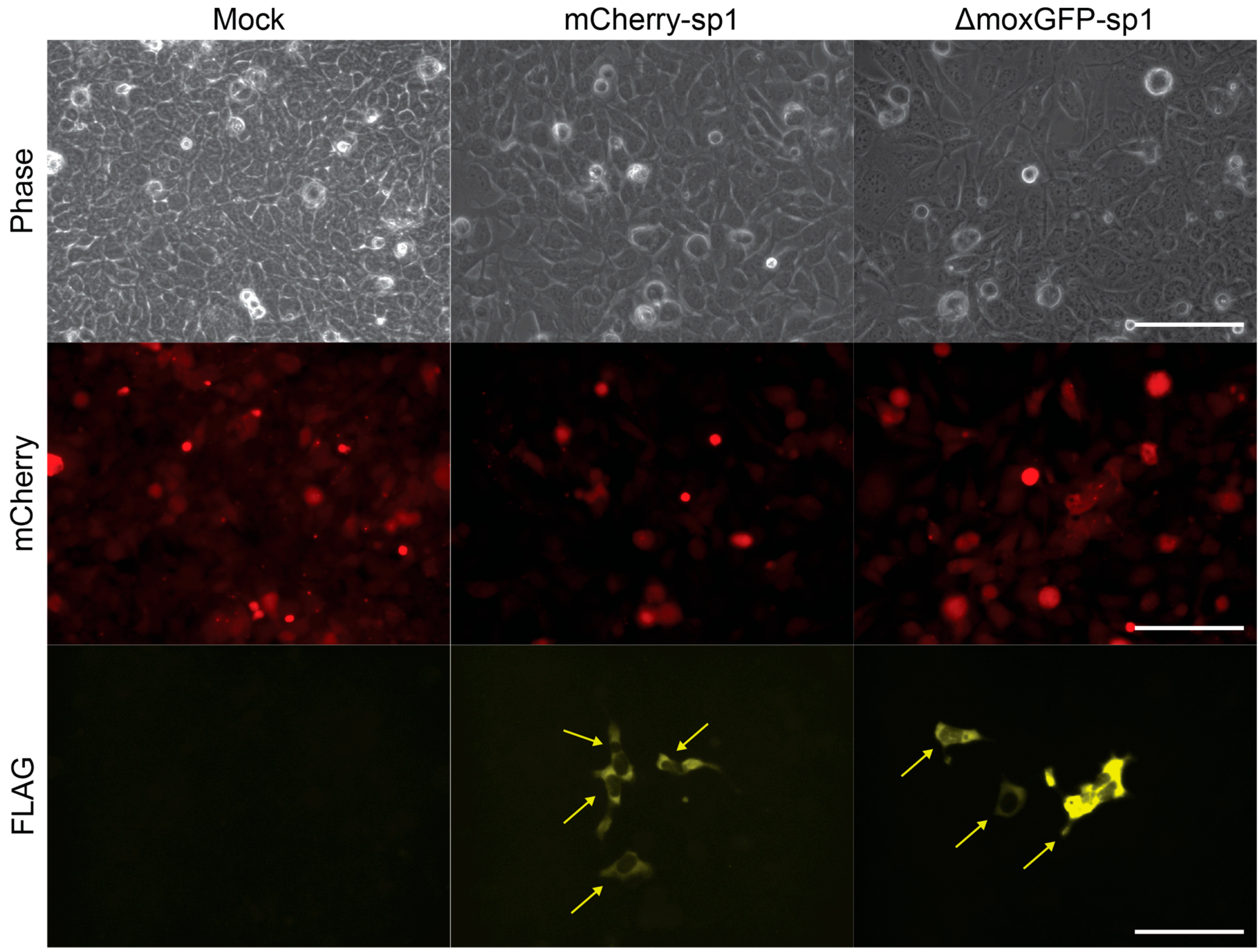

Figure S1. FLAG staining of edited BETLE-Pop. BETLE-Pop cells were transfected with spCas9 and gRNAs targeting either mCherry or ΔmoxGFP. Cells were stained using an anti-FLAG antibody and visualized via fluorescence microscopy; scale bars, 100 μm.

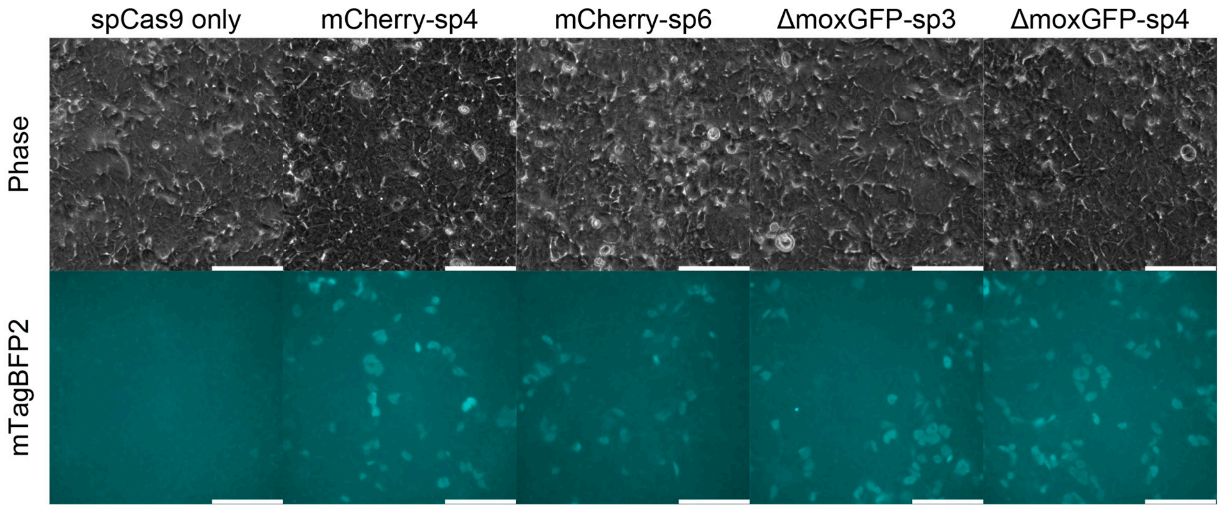

Figure S2. Spatial profile of edited BETLE-Pop expressing mTagBFP2. BETLE-Pop cells were transfected with SpCas9 and gRNAs targeting either mCherry or ΔmoxGFP. BETLE reporter editing and subsequent mTagBFP2 expression was monitored via fluorescence microscopy; scale bars, 100 μm.

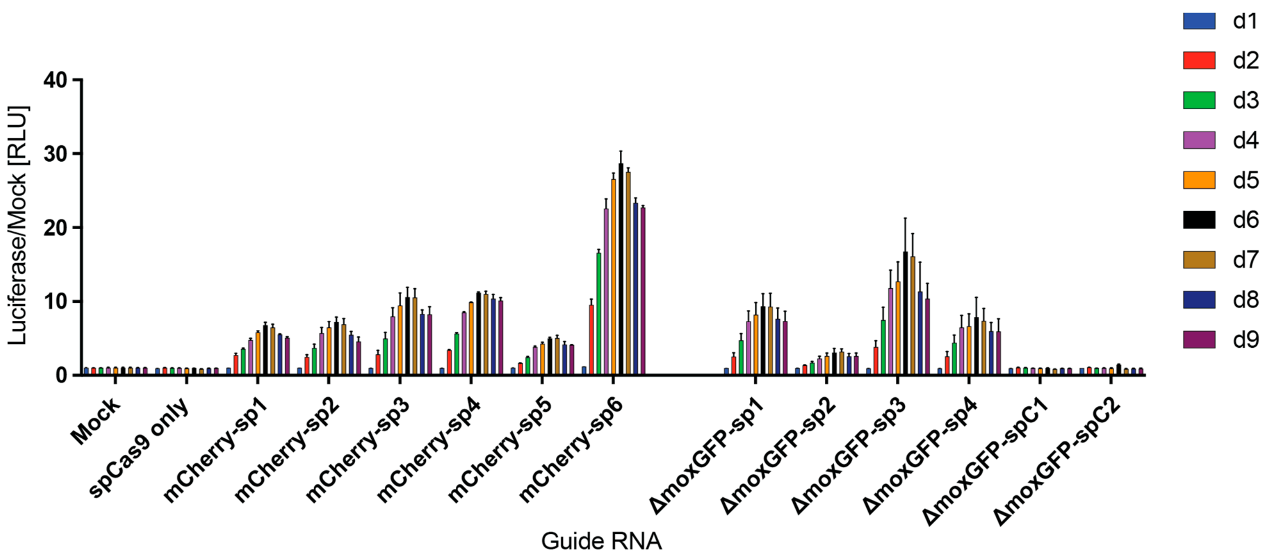

Figure S3. Editing-mediated NanoLuc luciferase expression kinetics. BETLE-Pop cells were transfected with SpCas9 and guide RNAs. Luciferase activity in the supernatant of transfected cells was measured every 24h for 9 days.

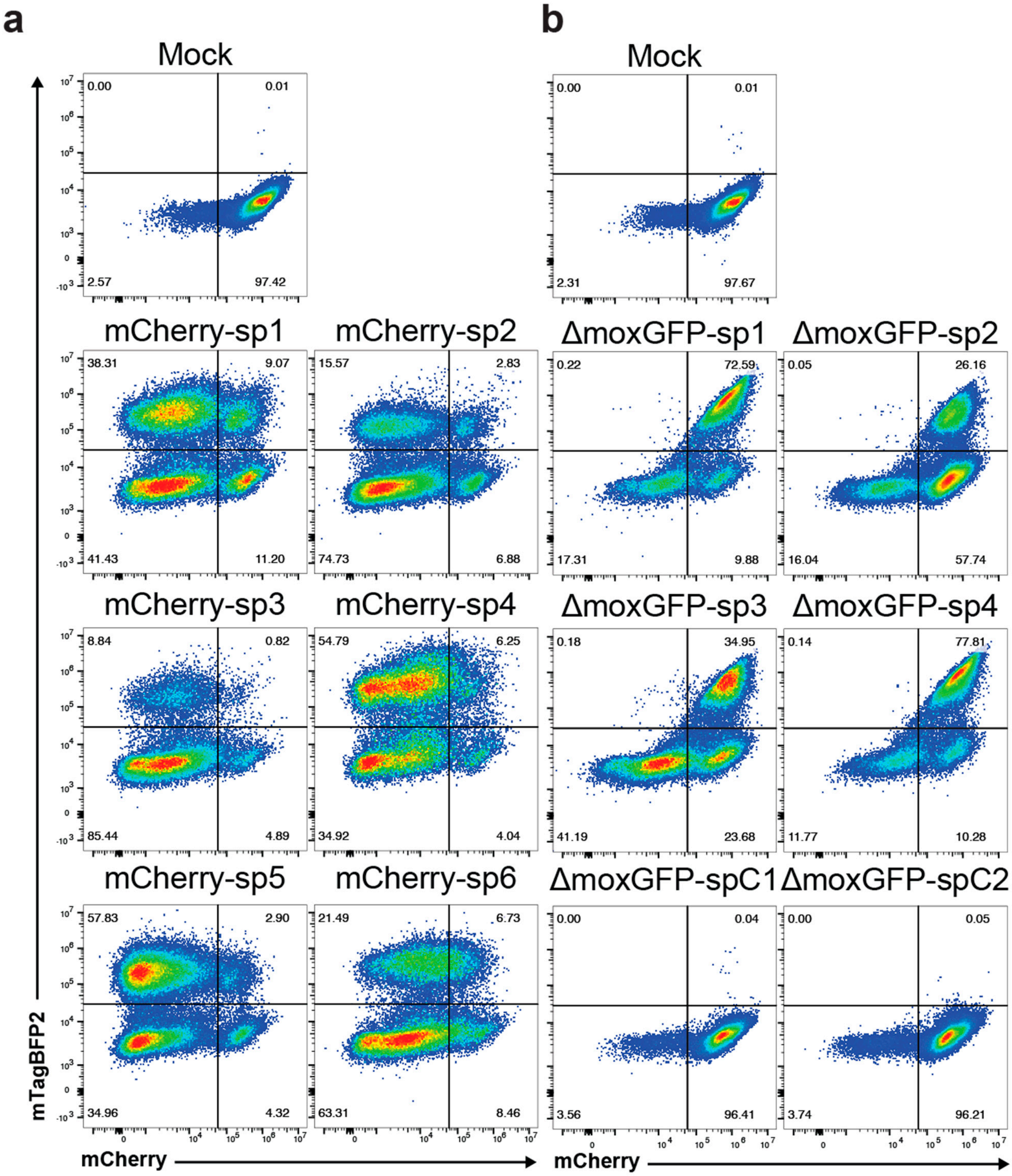

Figure S4. Puromycin selection of Cas9 edited cells. BETLE-Pop cells were transfected with SpCas9 and guide RNAs. Twenty-four hours later 100 µg/mL puromycin was added to the culture media for 48 hours. BETLE reporter editing was analyzed via FACS analysis.

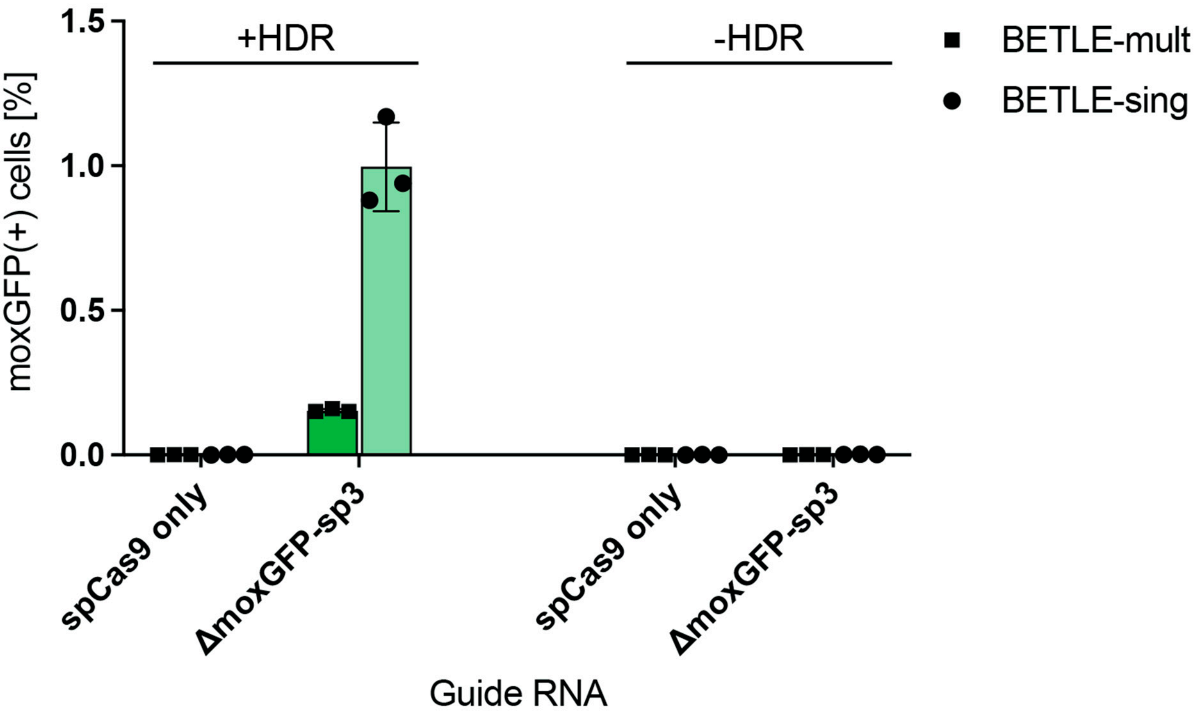

Figure S5. HDR efficiency in BETLE-mult and BETLE-sing cells. BETLE-mult and BETLE-sing cells were co-transfected with gRNA ΔmoxGFP-sp3 with and without HDR template and the percentage of moxGFP(+) cells assessed by flow cytometry.

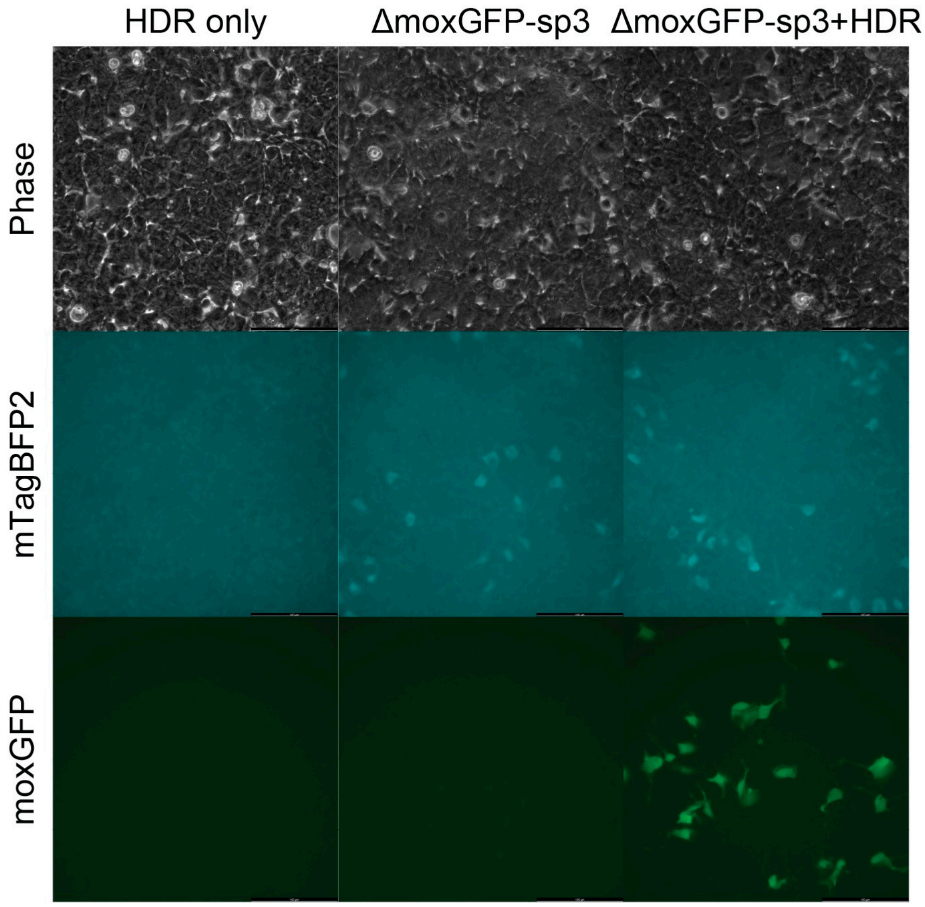

Figure S6. HDR-repair of ΔmoxGFP in BETLE-sing cells. BETLE-sing cells were co-transfected with gRNA ΔmoxGFP-sp3 with and without HDR template. Fluorescent reporter expression was monitored by fluorescence microscopy; scale bars, 100 μm.

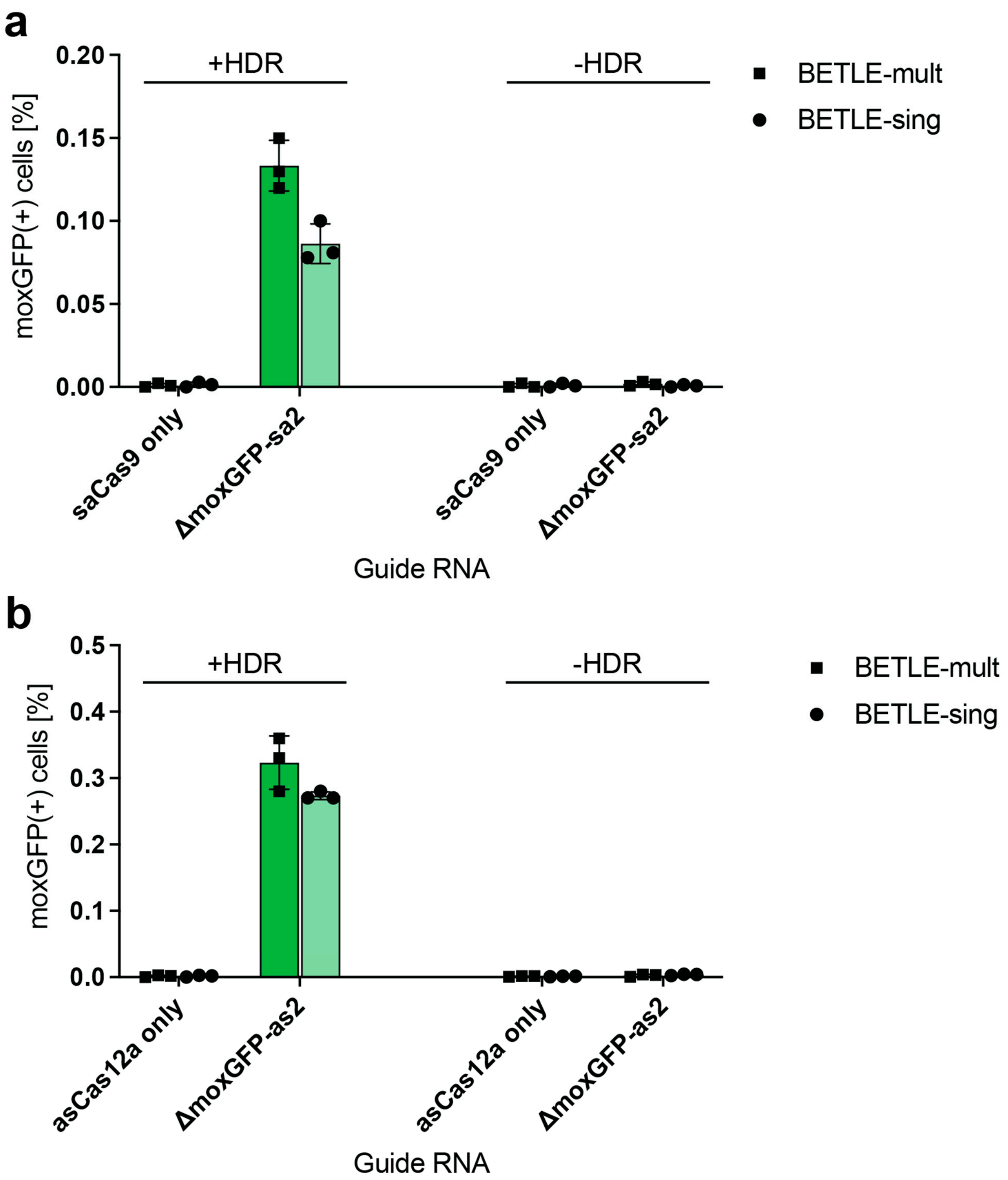

Figure S7. HDR efficiency in BETLE-mult and BETLE-sing cells. BETLE-mult and BETLE-sing cells were a) co-transfected with gRNA ΔmoxGFP-sa2 with and without HDR template or b) co-transfected with gRNA ΔmoxGFP-as2 with and without HDR template. The percentage of moxGFP(+) cells was assessed by flow cytometry.

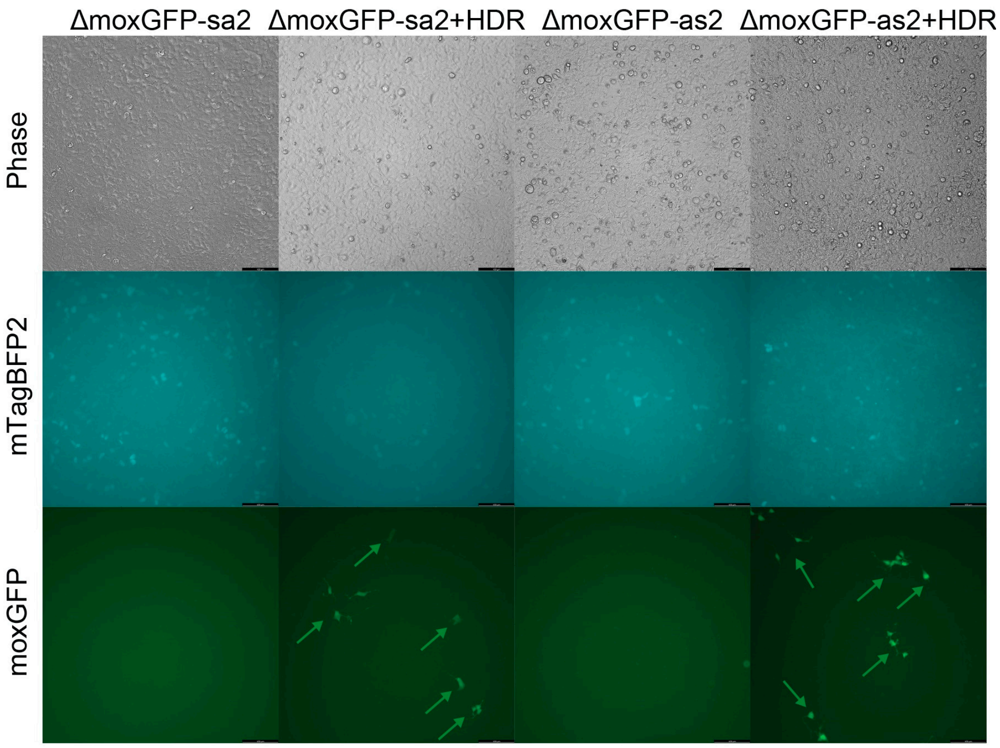

Figure S8. HDR-repair of ΔmoxGFP using saCas9 and asCas12a. BETLE-sing cells were transfected with saCas9 or asCas12a gRNAs with and without HDR template. Fluorescent reporter expression was monitored by fluorescence microscopy; scale bars, 100 μm.

Table S1. Sequences of CRISPR-Cas gRNAs utilized in the study. Mismatches are marked in lower-case.

Reporter site	Cas enzyme	Guide name	Sequence
A	spCas9	mCherry-sp1	CTGTCCCCTCATTCATGTA
	spCas9	mCherry-sp2	TCAGTTCATGTACGGCTCCA
	spCas9	mCherry-sp3	GTCGGCGGGGTGCTTGACGT
B	spCas9	mCherry-sp4	CTCGGGGTACATCCGCTCGG
C	spCas9	mCherry-sp5	GCGTTCGTACTGTTCCACGA
	spCas9	mCherry-sp6	GAACAGTACGAACGCGCCGA
D	spCas9	ΔmoxGFP-sp1	TGGTCACCACCCTCACAAAG
	spCas9	ΔmoxGFP-sp2	GTCGTGCCGCTTTGTGAGGG
	spCas9	ΔmoxGFP-sp3	GAAGTCGTGCCGCTTTGTGA
	spCas9	ΔmoxGFP-sp4	AGAAGTCGTGCCGCTTTGTG
	spCas9	ΔmoxGFP-spC1	TTCAAGAGcGCCATGCCcGA
	spCas9	ΔmoxGFP-spC2	TGGCGcTCTTGAAGAAGTCG
	saCas9	ΔmoxGFP-sa1	GAAGAAGTCGTGCCGCTTTGT
	saCas9	ΔmoxGFP-sa2	ACAAAGCGGCACGACTTCTTC
	saCas9	ΔmoxGFP-saC1	GAcGAAtgCGTGCCGCTTTGT
	asCas12a	ΔmoxGFP-as1	TGAGGGTGGTGACCAAAGTGGGC
	asCas12a	ΔmoxGFP-as2	GTCACCACCCTCACAAAGCGGCA
	asCas12a	ΔmoxGFP-as3	GACAGCTTGGACTGGGTGGACAG
	asCas12a	ΔmoxGFP-asC1	GTCtCCACCagCACAAAGCGGCA

Table S2. Information on BETLE reporter integration sites for BETLE-mult and BETLE-sing.

Sample	**Chromosome**	**Orientation**	**Position**	**% of total reads**
BETLE-Mult	1	minus	206276044	10.81
BETLE-Mult	1	plus	225589572	29.73
BETLE-Mult	6	minus	64647493	18.92
BETLE-Mult	8	plus	17767389	10.81
BETLE-Mult	10	plus	33291020	18.92
BETLE-Sing	20	minus	53753872	100

Table S3. Primers used for esTaq-PCR.

Name	**Purpose**	**Sequence**
PB-3TR-Outer	piggyBac Integration, PCR1	GCGACGGATTCGCGCTATTT
PB-5TR-Outer	piggyBac Integration, PCR1	GACCGA-TAAAACACATGCGTCA
PB-3TR-Inner-P5-TruSeq-R1	piggyBac Integration, PCR2	AATGATACGGCGACCAC-CGAGATCTACAC-TCTTTCCCTACACGAC-GCTCTTCCGATCTATTTCAAGAATGCATGCGTCA
PB-5TR-Inner-P5-TruSeq-R1	piggyBac Integration, PCR2	AATGATACGGCGACCAC-CGAGATCTACAC-TCTTTCCCTACACGAC-GCTCTTCCGATCTCACATGATTATCTTTAACGTACGTCAC
P7-N721-NexteraR2	Nested/Index PCR	CAAGCAGAAGACGGCAT-ACGAGATGCAGCGTAG-TCTCGTGGGCTCGGAGATG
P7-N722-NexteraR2	Nested/Index PCR	CAAGCAGAAGACGGCAT-ACGAGATCTGCG-CATGTCTCGTGGGCTCGGA-GATG
P7-N723-NexteraR2	Nested/Index PCR	CAAGCAGAAGACGGCAT-ACGAGATGAGCGCTAG-TCTCGTGGGCTCGGAGATG
P7-N724-NexteraR2	Nested/Index PCR	CAAGCAGAAGACGGCAT-ACGAGATCGCTCAG-TGTCTCGTGGGCTCGGA-GATG
P7-N725-NexteraR2	Nested/Index PCR	CAAGCAGAAGACGGCAT-ACGA-GATGTCTTAGGGTCTCGTGGGCTCGGAGATG
P7-N726-NexteraR2	Nested/Index PCR	CAAGCAGAAGACGGCAT-ACGAGA-TACTGATCGGTCTCGTGGGCTCGGAGATG
P7-N727-NexteraR2	Nested/Index PCR	CAAGCAGAAGACGGCAT-ACGAGA-TACTGATCGGTCTCGTGGGCTCGGAGATG
P7-N728-NexteraR2	Nested/Index PCR	CAAGCAGAAGACGGCAT-ACGAGATTAGCTGCAG-TCTCGTGGGCTCGGAGATG
P7-N729-NexteraR2	Nested/Index PCR	CAAGCAGAAGACGGCAT-ACGAGATGACGTCGAG-TCTCGTGGGCTCGGAGATG
